# Intraoperative Methadone for Post-craniotomy Pain Control: A Matched Cohort Exploratory Framework Study

**DOI:** 10.7759/cureus.102846

**Published:** 2026-02-02

**Authors:** Zoey A Croft, Sean Inzerillo, Shoaib Syed, Laura Mittelman, Ryan McCann, Samuel Latzman, Aryaa Karkare, Jared Bassett, Jason Burian, Brianna Suffren, Priscilla Nelson, Randy D'Amico

**Affiliations:** 1 Neurosurgery, Northwell Health Lenox Hill Hospital, New York, USA; 2 Neurosurgery, State University of New York (SUNY) Downstate Health Sciences University, Brooklyn, USA; 3 Neurosurgery, Donald and Barbara Zucker School of Medicine at Hofstra/Northwell, Hempstead, USA; 4 Neurosurgery, Donald and Barbara Zucker School of Medicine at Hofstra/Northwell, New York, USA; 5 Anesthesiology, Northwell Health Lenox Hill Hospital, New York, USA

**Keywords:** craniotomy, methadone, opioid agonist, pain management, postoperative pain

## Abstract

Background

Postoperative pain following craniotomy is commonly treated with multimodal analgesic regimens requiring frequent dosing, which contributes to an increased opioid burden. Methadone, a μ-opioid receptor agonist and N-methyl-D-aspartate (NMDA) receptor antagonist, may provide extended analgesia in craniotomy patients from a single intraoperative dose.

Methods

We performed a single-center feasibility retrospective matched cohort study of adult patients undergoing craniotomy for tumor resection between October 2024 and March 2025. Primary outcomes included postoperative pain scores and opioid consumption at 24, 48, and 72 hours.

Results

Thirty-two patients (eight methadone, 24 controls) were analyzed. Baseline demographics and perioperative characteristics were comparable. At 24 hours, pain scores and opioid use were similar between groups. At 48 and 72 hours, the methadone group demonstrated numerically lower pain scores and opioid consumption, though the study was underpowered to interpret these differences. No methadone-associated safety concerns were identified.

Conclusions

Intraoperative methadone was feasible and appeared comparable to conventional opioid regimens, with trends toward reduced pain and opioid use at later timepoints. These exploratory findings support further prospective trials to clarify the role of methadone in neurosurgical analgesia.

## Introduction

Acute pain following craniotomy is common, reported in up to 90% of patients in the immediate perioperative period, yet remains variably recognized and undertreated [1,2]. Early perceptions that craniotomy is not associated with significant pain, coupled with longstanding concerns that systemic opioid use might obscure neurologic assessment, cause respiratory depression, or elevate intracranial pressure, have contributed to a conservative approach to analgesia in neurosurgical patients [3]. Nevertheless, nociceptive input from the scalp, dura, periosteum, and associated musculature produces meaningful pain. Inadequately controlled acute pain has been linked to impaired recovery and a substantial risk of chronic postsurgical pain [1,2,4-6].

Current multimodal analgesic strategies, including acetaminophen, non-steroidal anti-inflammatory drugs (NSAIDs), local anesthetics (e.g., scalp blocks), and short-acting opioids such as fentanyl, are inconsistently applied and often require frequent redosing, which can increase cumulative opioid exposure. Thus, there remains a need for safe, durable, and pragmatic analgesic strategies in patients undergoing craniotomy.

Methadone is a synthetic μ-opioid receptor agonist with additional N-methyl-D-aspartate (NMDA) receptor antagonist activity [7-9]. This dual mechanism may reduce central sensitization and opioid-induced hyperalgesia, offering both intraoperative and extended postoperative analgesia [5,8,10]. In contrast to short-acting opioids commonly used during craniotomy, methadone has a prolonged and variable half-life, with clinically meaningful analgesic effects persisting well beyond the immediate intraoperative period and that of other clinically used opioids [5]. Methadone also differs from other intraoperative opioids in that its clinical analgesic duration may approximate its elimination half-life when administered at sufficient doses, allowing a single intraoperative dose to provide stable analgesia throughout the period of peak postoperative pain [11]. Prior literature has demonstrated that methadone’s analgesic activity may continue for 24-36 hours or longer following a single dose, distinguishing it from conventional opioids that require frequent redosing [4,5,11,12]. This prolonged effect is particularly relevant in the perioperative setting, where methadone’s sustained receptor activity may reduce the need for repeated opioid administration during the first perioperative day [11]. Randomized and observational studies in non-neurosurgical populations, including bariatric, spine, cardiac, and otolaryngologic surgery, have shown that intraoperative methadone can improve pain control and reduce postoperative opioid requirements without excess complications [7,9,13].

Despite the evidence, methadone remains underutilized in neurosurgery. Concerns about delayed emergence from anesthesia, oversedation, QTc prolongation, and potential interference with early neurologic assessments have limited its adoption [1,5,14]. Few studies have specifically examined intraoperative methadone in craniotomy patients, and to our knowledge, none have systematically compared postoperative pain outcomes in this population using a matched cohort design.

We therefore performed an exploratory retrospective matched cohort study of patients undergoing elective craniotomy for tumor resection, comparing those who received intraoperative methadone with those who received conventional opioid regimens. We hypothesized that intraoperative methadone would be safe and that it would provide comparable or improved postoperative analgesia, thereby supporting future prospective evaluation of methadone in neurosurgical pain management.

## Materials and methods

We conducted a single-center retrospective matched cohort study under Institutional Review Board Approval (Protocol #21-0008). All adult patients undergoing supratentorial craniotomy for brain tumor resection were isolated from a de-identified departmental database between October 2024 and March 2025. This study was conducted in adherence with Strengthening the Reporting of Observational Studies in Epidemiology (STROBE) guidelines. 

**Figure 1:**
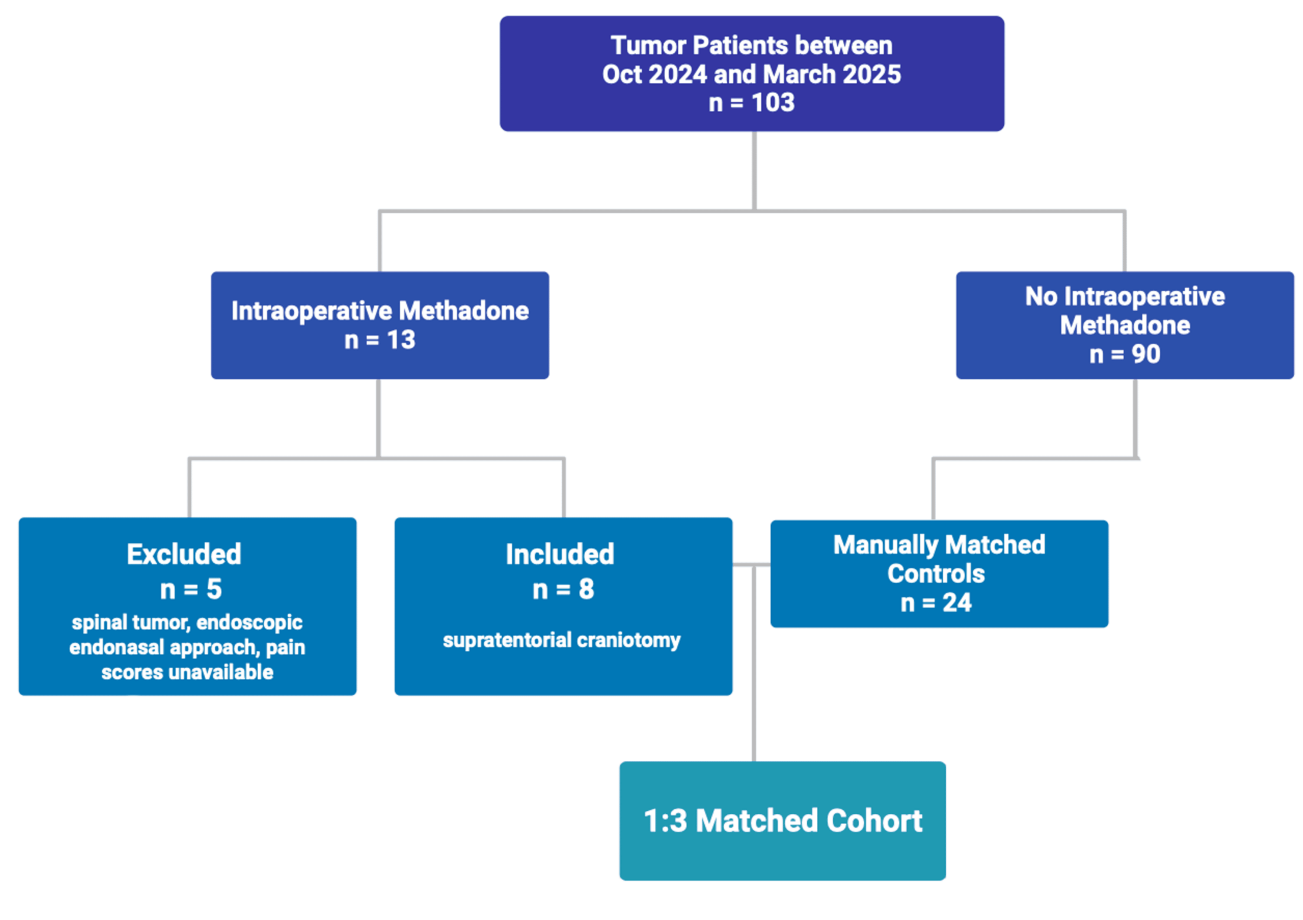
Flow diagram of patient enrollment and matching.

Patients were included if they had complete demographic, perioperative, and postoperative data available. Required outcomes included numeric pain scores (0-10 scale) and morphine equivalent opioid dosing at 24, 48, and 72 hours after surgery. Patients discharged before 48 or 72 hours were excluded from those respective timepoint analyses (Figure 1).

Patients were categorized into two groups: those receiving intraoperative methadone (methadone group) and those who did not receive intraoperative methadone (control group). Across both groups, anesthetic agents used for induction consisted of combinations of propofol, short-acting opioids (e.g., fentanyl), and neuromuscular blockers. Maintenance was primarily achieved using propofol. Adjunct medications administered intraoperatively included anticholinergics, corticosteroids, antibiotics, diuretics, and additional non-opioid analgesics (e.g., acetaminophen or NSAIDs) used as clinically indicated. In the methadone group, a single intravenous dose of 10-20 mg of methadone was administered intraoperatively at the discretion of the attending anesthesiologist.

To reduce baseline confounding, patients in the methadone group were matched to controls in a 1:3 ratio (Figure 1). Matching variables included age, sex, body mass index (BMI), American Society of Anesthesiologists (ASA) physical status, preoperative opioid use, surgery duration, and anesthesia duration. Matching was conducted using a direct comparison framework modeled after previously published pilot studies [15]. Investigators were blinded to postoperative outcomes during the matching process. This approach was used to maximize comparability within a small sample size.

The primary outcomes were postoperative pain scores and opioid consumption at 24, 48, and 72 hours. Pain was assessed using the 0-10 numeric pain rating scale (NPRS), documented routinely by nursing staff as the patient’s reported pain at rest, and each time opioids were administered, consistent with the standard of care. Opioid use was abstracted from medication administration records and converted to morphine milligram equivalents (MMEs) using standard opioid conversion factors [16].

Secondary outcomes included inpatient complications identified through review of the electronic medical record. Minor complications were defined as postoperative nausea/vomiting, transient urinary dysfunction, or mild electrolyte imbalances. Serious complications included events requiring escalation of care, including new-onset seizures, bleeding, or stroke. 

Statistical analysis

Continuous variables were presented as medians with interquartile ranges (IQR) and compared between groups using the Wilcoxon rank-sum test. Categorical variables were compared using Fisher’s exact test. Pain scores and opioid consumption were evaluated at each timepoint independently. In addition to hypothesis testing, between-group differences were summarized using the Hodges-Lehmann estimator, calculated as the median of all pairwise differences with 95% confidence intervals (CIs), to allow assessment of effect size. Statistical significance was defined as p < 0.05. Analyses and data visualization were performed using R version 4.5.1 (R Project for Statistical Computing, Vienna, Austria).

## Results

Cohort selection and baseline characteristics

During the study period, 60 patients underwent craniotomy for brain tumor resection. Eight patients (13%) received intraoperative methadone, and 52 (87%) did not. After 1:3 matching on prespecified variables, 32 patients were selected for analysis (methadone group n = 8; control group n = 24). Baseline characteristics were well balanced with no statistically significant between-group differences (Table 1). Median age was 54.0 years in the methadone group and 59.0 years in controls. Sex distribution, BMI, ASA class, preoperative opioid exposure, and operative/anesthetic durations were comparable.

**Table 1:** Baseline demographic and clinical characteristics of patients receiving intraoperative methadone versus controls. ASA = American Society of Anesthesiologists; BMI = body mass index; IQR = interquartile range

Variable	Control (n = 24)	Methadone (n = 8)	p-value
Age, median (IQR)	59.0 (42.0-66.2)	54.0 (32.5-67.2)	0.913
Male, n (%)	12 (50.0%)	4 (50.0%)	1.000
BMI, median (IQR)	26.4 (25.1-29.4)	23.9 (23.1-26.5)	0.151
ASA score, median (IQR)	3 (3-3)	3 (3-3)	0.763
Pre-op opioid use, n (%)	13 (54.2%)	3 (37.5%)	0.685
Surgery duration, min (IQR)	184 (156-246)	174 (154-220)	0.965
Anesthesia duration, min (IQR)	292 (272-384)	276 (242-342)	0.454

Given the variation in opioid conversion charts available, dosing and conversion factors for the methadone group at 24, 48, and 72 hours have been provided (Table 2) [16].

**Table 2:** Methadone and additional post-operative opioid dosing with MME conversions. Opioid type remained consistent in patients with 48-hour and 72-hour dosages. *10 mg vs. 20 mg methadone dosing was based on a BMI over 26.

Subject (methadone)	Methadone dose*	Drug (24 hours)	Dose (24 hours)	Conversion factor	MME (24 hours)	MME (48 hours)	MME (72 hours)
P1	10 mg	Fentanyl IV	12.5ug	0.1	1.25	0	Discharged
P2	10 mg	Oxycodone PO	10 mg	1.5	15	0	Discharged
P3	10 mg	Oxycodone PO	10 mg	1.5	15	0	0
P4	20 mg	Oxycodone PO, Hydromorphone IV	10 mg, 0.5mg	1.5, 20	25	0	0
P5	10 mg	Oxycodone PO	10 mg	1.5	15	22.5	Discharged
P6	10 mg	Oxycodone PO	15 mg	1.5	22.5	0	0
P7	20 mg	Oxycodone PO	10 mg	1.5	15	0	Discharged
P8	20 mg	Oxycodone PO	15 mg	1.5	22.5	15	22.5

Primary outcomes: pain and opioid consumption

**Figure 2:**
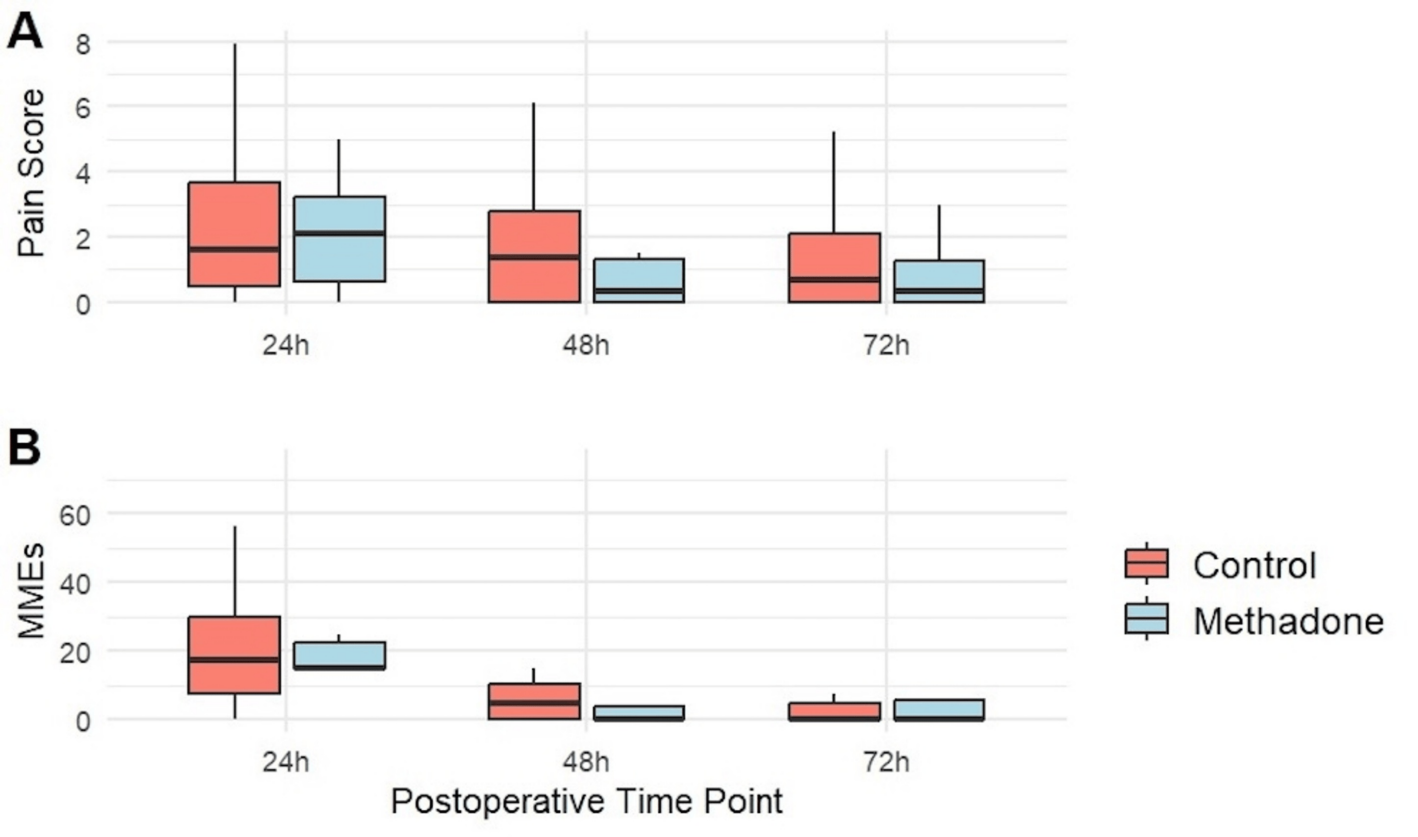
Postoperative pain scores (A) and opioid consumption (B) at 24, 48, and 72 hours in the methadone and control groups. MME = morphine milligram equivalent

Pain scores (0-10 NPRS at rest) and opioid consumption (MME) were evaluated at 24, 48, and 72 hours postoperatively (Table 3, Figure 2). At 24 hours after surgery, pain scores (median difference 0.0; 95% CI -1.6-1.6; p = 1.000) and opioid use (median diff. 0.0 MME; 95% CI -13.7-7.5; p = 0.861) were comparable between groups.

**Table 3:** Comparison of postoperative pain scores and opioid consumption by group at 24, 48, and 72 hours. CI = confidence interval; HL = Hodges-Lehmann; IQR = interquartile range; MME = morphine milligram equivalent *Excludes four methadone and four control patients discharged before the 72-hour timepoint.

Outcome	Group	Median (IQR)	HL median difference	HL 95% CI	p-value
Pain 24 hours	Control	1.6 (0.5-3.7)	0.0	-1.6-1.6	1.000
	Methadone	2.1 (0.6-3.2)			
MME 24 hours	Control	17.5 (7.5-30.0)	0.0	-13.7-7.5	0.861
	Methadone	15.0 (15.0-22.5)			
Pain 48 hours	Control	1.4 (0.0-2.8)	-0.6	-2.0-0.3	0.266
	Methadone	0.3 (0.0-1.3)			
MME 48 hours	Control	5.0 (0.0-10.3)	0.0	-7.5-0.0	0.192
	Methadone	0.0 (0.0-3.8)			
Pain 72 hours^*^	Control	0.7 (0.0-2.1)	0.0	-2.4-1.0	0.658
	Methadone	0.3 (0.0-1.3)			
MME 72 hours^*^	Control	0.0 (0.0-5.0)	0.0	-5.0-17.5	0.603
	Methadone	0.0 (0.0-5.6)			

By 48 hours after surgery, the methadone group demonstrated numerically lower pain scores (median difference -0.6; 95% CI -2.0-0.3; p = 0.266) and comparable opioid requirements (median difference 0.0 MME; 95% CI -7.-0.0; p = 0.192). At 72 hours, pain scores (median difference 0.0; 95% CI -2.4-1.0; p = 0.658) and opioid use (median diff. 0.0 MME; 95% CI -5.0, 1.5; p = 0.603) were comparable between groups. Notably, four patients from each group were discharged before 72 hours and were excluded from 72-hour analyses. 

Given the small sample, between-group differences did not reach statistical significance at any timepoint; however, the direction of effect favored methadone at 48 hours for both outcomes. 

Complications (secondary outcomes)

Inpatient minor complications occurred in 25.0% of patients in each group. Serious complications were observed in 0.0% of methadone patients and 25.0% of control patients. Serious complications in the control group included intraparenchymal hemorrhage (n = 2), seizure (n = 1), ischemic stroke (n = 1), pneumocephalus (n = 1), and a failed swallow test resulting in nasogastric intubation (n = 1).

Sensitivity and missingness considerations

Timepoint analyses reflect available case data at each interval; early discharge led to expected missingness at 72 hours. Because pain scores and MME opioid dosing were assessed at fixed cross-sectional timepoints rather than longitudinally modeled, results should be interpreted as exploratory signals rather than definitive estimates of treatment effect.

## Discussion

Summary of findings

In this exploratory matched cohort study, intraoperative methadone administration was associated with pain and opioid consumption profiles comparable to conventional regimens after craniotomy. While differences at 48 hours favored methadone, the limited sample size of eight patients in the methadone group rendered the analysis underpowered. However, analysis of the effect size through postoperative median differences and respective confidence intervals provides context by highlighting the directionality of outcomes, which point to decreased pain and opioid consumption, especially at 48 hours. These data suggest that methadone can reliably be incorporated into anesthetic protocols for selected patients, but definitive conclusions cannot be drawn. 

Comparison to prior literature

Our findings are consistent with prior studies demonstrating that intraoperative methadone may extend analgesia and reduce postoperative opioid requirements in patients without cranial pathology, including spine, cardiac, bariatric, and tonsillectomy cohorts [9,13,17-21]. In a meta-analysis of 382 patients across surgical specialties, methadone administration was associated with significantly lower opioid post-operative opioid consumption (mean difference -15.22 MME; p = 0.01), and seven of the ten included studies reported lower pain scores both at rest and with movement [22]. Similarly, a double-blinded, dose-finding pilot study in 60 same-day ambulatory surgical patients demonstrated that intraoperative methadone significantly reduced opioid consumption after discharge compared with conventional regimens (p < 0.01) [23]. These studies highlight the interdisciplinary role of methadone in reproducing analgesic benefit and provide important context for its evaluation in neurosurgical populations. To our knowledge, this is the first study to apply a structured matched cohort framework in craniotomy patients, addressing the gap in the neurosurgical analgesia literature.

Considerations in interpreting MME for long-acting opioids

Interpretation of postoperative opioid consumption using MMEs warrants careful consideration in the context of methadone. MME provides a standardized estimate of relative opioid potency at the time of administration but does not account for differences in pharmacokinetics or duration of analgesic effect among opioids [24]. This limitation is particularly relevant for methadone, whose half-life results in sustained opioid receptor activity after a single dose [11]. Consequently, MME-based comparisons may underestimate the cumulative analgesic contribution of methadone relative to short-acting opioids that require repeated dosing to maintain analgesia [11]. This distinction proves important context for interpreting opioid consumption patterns in the present study and supports cautious interpretation of MME-based comparison when evaluating long-acting opioids. 

Safety considerations

No methadone-specific adverse events were identified, and the frequency of minor complications was similar between groups. Although serious complications occurred only in controls, the small methadone group sample (n = 8) precludes meaningful safety comparisons and should be interpreted cautiously. Given methadone’s pharmacology, vigilance remains warranted: QTc prolongation, delayed respiratory depression, and oversedation remain theoretical risks. Careful patient selection, avoidance of concomitant QT-prolonging medications, and standardized postoperative monitoring are prudent safeguards [8,25,26]. These findings are consistent with larger perioperative series that report a low incidence of serious adverse events with a single intraoperative dose of methadone [8,25,26].

Clinical implications

From a practical standpoint, a single intraoperative dose of methadone may provide stable analgesia across the first 48-72 hours after craniotomy, potentially reducing the need for frequent redosing of short-acting opioids. This could be particularly advantageous in neurosurgical patients, where minimizing sedation facilitates early and reliable neurologic assessments. However, given that the current data are underpowered, methadone use in this context should be considered a potential investigational adjunct rather than a practice standard in neurosurgical anesthesia.

Limitations

This study has several limitations. As discussed in our interpretation, the small sample size limits power and precision. Additionally, despite efforts to balance covariates, the matching process retains the potential to introduce subjectivity. The specific location of the supratentorial craniotomy was not controlled for, and neither was tumor pathology. Retrospective pain assessments are subject to variability in nursing documentation and patient perception. Inpatient opioid administration was prescribed as-needed and could be influenced by provider discretion or institutional habits. The concurrent use of additional intraoperative analgesics may have influenced postoperative pain trajectories and opioid requirements. These agents were administered consistently across groups according to routine clinical practice rather than a standardized protocol, which introduces variability that cannot be fully controlled in a retrospective design. Finally, because pain scores and MME opioid dosing were analyzed at fixed timepoints, we could not capture longitudinal dynamics of the pain trajectory.

The lack of statistical power in this study, the short timeframe of retrospective review, and confounders limit the ability to detect modest effect sizes, and the findings should therefore be interpreted as preliminary and intended to generate hypotheses for future adequately powered prospective studies rather than to establish definitive clinical conclusions.

Future directions

Though these findings cannot provide clinical guidance, they provide a rationale for studying methadone in craniotomy patients and a foundation for conducting prospective studies and randomized controlled trials with formal feasibility endpoints. Future work should evaluate expanded and prospective cohorts, explore optimal dosing, incorporate safety monitoring protocols, and integrate this workflow within enhanced recovery pathways. Larger datasets will also be necessary to rigorously assess safety outcomes and clarify whether methadone meaningfully reduces opioid burden in neurosurgery.

## Conclusions

In this exploratory matched cohort, a single intraoperative dose of methadone appeared comparable to conventional opioid regimens for post-craniotomy analgesia. While underpowered, trends toward lower pain scores and reduced opioid use at 48-72 hours warrant further investigation. No safety signal attributable to methadone was identified, but larger prospective studies are needed to define its efficacy, safety, and role in multimodal neurosurgical analgesia.
